# Abnormal Behaviors and Developmental Disorder of Hippocampus in *Zinc Finger Protein 521 (ZFP521)* Mutant Mice

**DOI:** 10.1371/journal.pone.0092848

**Published:** 2014-03-27

**Authors:** Nobutaka Ohkubo, Etsuko Matsubara, Jun Yamanouchi, Rie Akazawa, Mamoru Aoto, Yoji Suzuki, Ikuya Sakai, Takaya Abe, Hiroshi Kiyonari, Seiji Matsuda, Masaki Yasukawa, Noriaki Mitsuda

**Affiliations:** 1 Department of Circulatory Physiology, Ehime University, Shitsukawa, Toon, Ehime, Japan; 2 Department of Hematology, Clinical Immunology and Infectious Diseases, Graduate School of Medicine, Ehime University, Shitsukawa, Toon, Ehime, Japan; 3 Department of Anatomy and Embryology, Graduate School of Medicine, Ehime University, Shitsukawa, Toon, Ehime, Japan; 4 Department of Pathophysiology, College of Pharmaceutical Sciences, School of Clinical Pharmacy, Matsuyama University, Matsuyama, Ehime, Japan; 5 Laboratory for Animal Resources and Genetic Engineering, RIKEN Center for Developmental Biology, Kobe, Hyogo, Japan; University of Nebraska Medical Center, United States of America

## Abstract

Zinc finger protein 521 (ZFP521) regulates a number of cellular processes in a wide range of tissues, such as osteoblast formation and adipose commitment and differentiation. In the field of neurobiology, it is reported to be an essential factor for transition of epiblast stem cells into neural progenitors *in vitro*. However, the role of *ZFP521* in the brain *in vivo* still remains elusive. To elucidate the role of ZFP521 in the mouse brain, we generated mice lacking exon 4 of the *ZFP521* gene. The birth ratio of our *ZFP521* ^Δ/Δ^ mice was consistent with Mendel's laws. Although *ZFP521* ^Δ/Δ^ pups had no apparent defect in the body and were indistinguishable from *ZFP521^+/+^* and *ZFP521* ^+/Δ^ littermates at the time of birth, *ZFP521* ^Δ/Δ^ mice displayed significant weight reduction as they grew, and most of them died before 10 weeks of age. They displayed abnormal behavior, such as hyper-locomotion, lower anxiety and impaired learning, which correspond to the symptoms of schizophrenia. The border of the granular cell layer of the dentate gyrus in the hippocampus of the mice was indistinct and granular neurons were reduced in number. Furthermore, Sox1-positive neural progenitor cells in the dentate gyrus and cerebellum were significantly reduced in number. Taken together, these findings indicate that ZFP521 directly or indirectly affects the formation of the neuronal cell layers of the dentate gyrus in the hippocampus, and thus *ZFP521* ^Δ/Δ^ mice displayed schizophrenia-relevant symptoms. *ZFP521* ^Δ/Δ^ mice may be a useful research tool as an animal model of schizophrenia.

## Introduction

ZFP521/Evi-3 protein in the mouse, also known as ZNF521/EHZF in humans, is a 1311 amino-acid-long nuclear factor that contains a putative nuclear localization signal, a nuclear remodeling and histone deacetylation complex (NuRD), and 30 zinc-finger motifs (ZF). The 6152 nucleotide-long mRNA comprises eight exons. Among them, exon 4 is the longest, consisting of 3353 nucleotides (approximately 85%), and encodes the motifs from ZF2 to ZF30 [1].

ZFP521 regulates the differentiation of several kinds of stem cells [2]–[4]. In CD34^+^ human hematopoietic progenitor cells, it suppresses their erythroid differentiation by inhibiting the transcriptional activity of hematopoietic transcriptional factor GATA-1 [5]. In mesenchymal stem cells, it suppresses their adipogenic determination and differentiation by binding to early B cell factor 1 (Ebf1) and inhibiting the expression of zinc finger protein 423 (ZFP423) [6], while it induces their osteogenic differentiation by inhibiting Runt related protein 2 (Runx2) [7]–[9].

In the field of neurobiology, Kamiya, *et al.* reported that ZFP521 is an essential factor for transition of epiblast stem cells into neural progenitors *in vitro*. They demonstrated that forced expression of ZFP521 leads to the neural conversion of embryonic stem (ES) cells even in the presence of bone morphogenetic protein 4 (BMP4); conversely, deprivation of ZFP521 by short hairpin RNA (shRNA) tends to halt the cell at the epiblast stage. The first eight zinc-finger motifs, from ZF1 to ZF8, were found to be essential for the neuralizing activity of ZFP521, because it triggers the neural differentiation of ES cells by associating with transcriptional co-activator p300 through these motifs. Also, ZF9 to ZF30 were found to be essential for this activity, because deletion of the motifs resulted in loss of activity [10]. ZF26 to ZF30 are required for interaction and inhibition of early B-cell factor 1 (EBF1), which is important for the development of striatonigral medium spiny neurons [11], [12]. However, the role of ZFP521 in the brain *in vivo* still remains elusive.

To elucidate the role of *ZFP521* in the mouse brain, we generated mice lacking exon 4 of the *ZFP521* gene, and analyzed them in detail.

## Materials and Methods

### Generation of ZFP521 Mutant Mice

**Figure 1:**
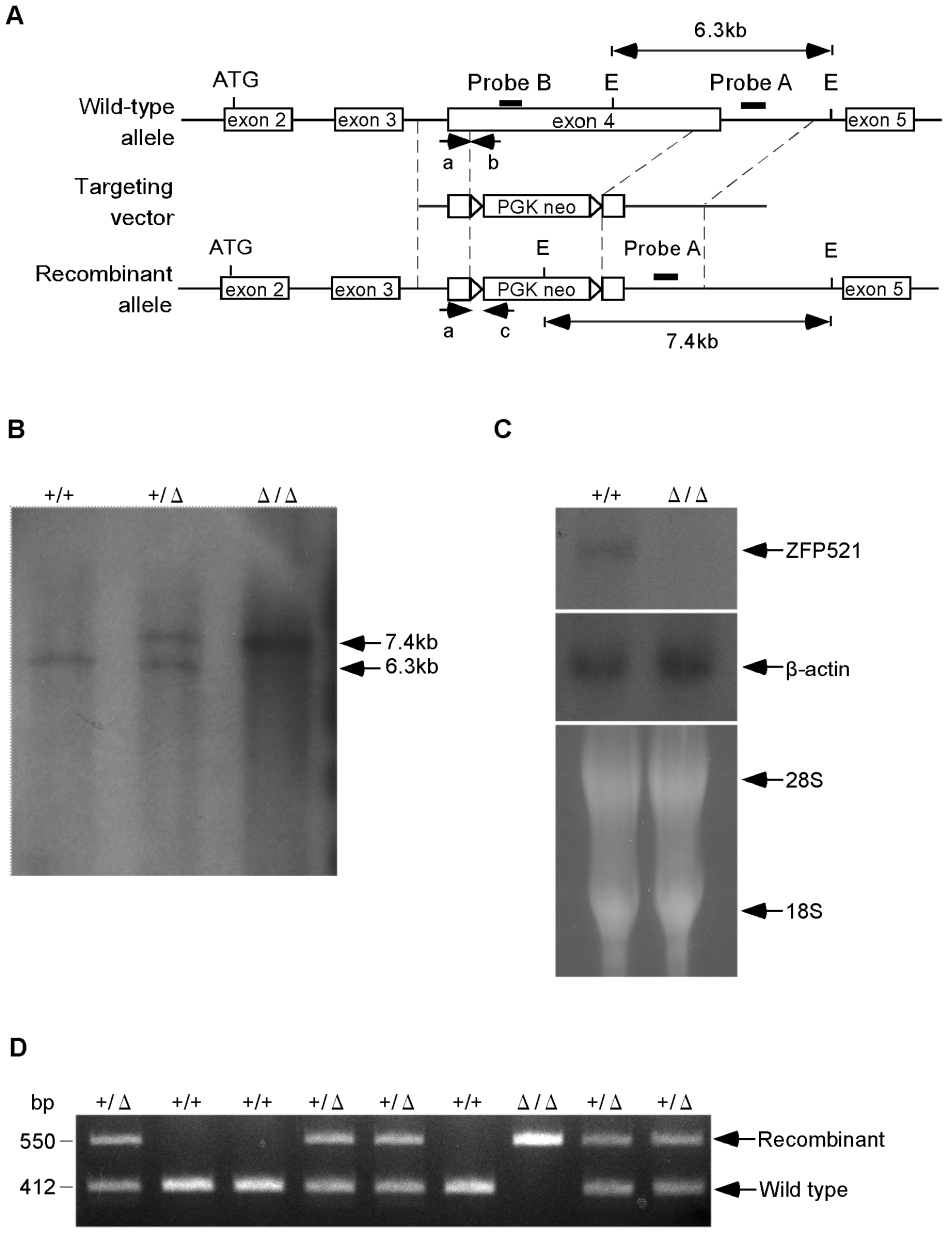
Disruption of *ZFP521* gene. (A) Structure of the wild-type allele of the *ZFP521* gene, targeting vector, and recombinant allele. E, EcoRI restriction sites; closed box, location of probe used for Southern blotting (Probe A) and Northern blotting (Probe B); arrowheads a–c, PCR primers for genotyping the mice. (B) Southern blot analysis of offspring from *ZFP521^+/^* ^Δ^ mice. Genomic DNAs were digested with EcoRI, and then subjected to Southern blotting. The 7.4-kb band, recombinant allele; 6.3-kb band, wild-type allele. (C) Northern blot analysis of total mRNA. Total RNA was extracted from mouse brain, and subjected to northern blotting. Hybridization of blots with labeled cDNA probe for β-actin (middle panel), with 18S rRNA and 28S rRNA (bottom panel) used as controls for RNA loading. (D) PCR genotyping using mouse tail. Primers a and b were used for detection of the wild-type allele (412 bp), and a and c were used for the knockout allele (550 bp).

A genomic fragment containing exon 4 of mouse *ZFP521* from C57BL/6J mouse was used to construct the targeting vector. For positive selection, the fragment containing most of exon 4 was replaced with a loxP/PGK-Neo-pA/loxP cassette (Figure 1A). The targeting vector was electroporated into ES cells. The clones resistant to G418 were screened for homologous recombination by PCR and confirmed by Southern blot analysis. Chimeric mice were generated by injection of targeted ES cells into blastocysts, and they were mated with C57BL/6J mice to obtain heterozygous mutant mice. The mice were backcrossed onto C57BL/6J for more than 10 generations, and after that intercrossed to obtain homozygous *ZFP521* mutant mice (Acc. No. CDB0952K: http://www.cdb.riken.jp/arg/mutant%20mice%20list.html).

### Animals

All mice were housed in an SPF facility, with a twelve-hour light/dark cycle, and fed water and a pellet chow diet *ad libitum*. The protocol for *ZFP521* mutant mice studies was approved by the Institutional Review Board of Ehime University Graduate School of Medicine (Permit Number:05-SO-38-16). All animal studies were carried out in accordance with the guidelines of the Ehime University School of Medicine Committee on Animals. All surgery was performed under sodium pentobarbital anesthesia, and all efforts were made to minimize suffering. Humane endpoints were employed during all animal experiments. Any of these animals were monitored daily, weighed weekly and euthanized at the humane endpoint after dramatic weight loss indicative of severe illness that would progress to death.

### Southern Blot Analysis

Genomic DNA was prepared from the tail using a DNeasy kit (QIAGEN, Tokyo, Japan), digested with EcoRI restriction enzyme, electrophoresed through a 1.0% agarose gel and transferred to nylon membrane Hybond-N^+^ (GE Healthcare, New York, USA). Then, a 0.7-kb DNA probe (probe A in Figure 1A) was made by PCR, purified with a Gel Extraction Kit (QIAGEN) and ^32^P-labeled with a Random Primer DNA Labeling Kit Ver.2 (TaKaRa, Otsu, Japan). Hybridization was carried out according to the standard method [13].

### Northern Blot Analysis

Total RNA was extracted from mouse whole brain using Isogen II (Nippon-Gene, Tokyo, Japan) as described in the manufacturer's protocol. RNAs (40 μg/lane) were dissolved in sample buffer (50% formamide, 2.2 M formaldehyde, 10 mM EDTA, 1x MOPS solution) and heated to 98°C for denaturing. Denatured samples were electrophoresed through 1% agarose-MOPS gel and transferred to nylon membrane (GE Healthcare). The blotted membrane was hybridized with a ^32^P-labeled probe corresponding to the DNA sequence of *ZFP521* (probe B in Figure 1A) or *β-actin* [14], [15].

### Genotyping

Mice were genotyped by polymerase chain reaction (PCR) using genomic DNA extracted from the tail, using the primers ZFP521-a: 5′-CAGCACGTGTAATGATATGTTCCTA-3′ and ZFP521-b: 5′-GGTGTGAGTCTTTAAGTGGATCTTG-3′ for the wild-type allele, and ZFP521-a and ZFP521-c: 5′-AGAAAGCGAAGGAGCAAAGCTG-3′ for the targeted allele [16].

### Body and Organ Weights

The living mice were weighed once a week. All data were derived from the healthy mice. If necessary, mice were euthanized at the predetermined humane endpoint. Five-week-old mice were anesthetized, perfused with phosphate-buffered saline (PBS) and dissected. Each organ was removed from the mice and weighed. Data were normalized by body weight.

### Growth Plate Histological Examination and Safranin O Staining

Femurs from five-week-old mice were processed for paraffin embedding with decalcification as described by Correa et al., and cut into 10-μm sections [7]. The sections were deparaffinized, stained with safranin-O and analyzed under a BZ-9000 microscope (Keyence, Osaka, Japan). The thickness of the femoral growth plate was measured using BZ-H1C software (Keyence).

### Behavioral Assays

An open field test was used to estimate locomotor activity [17], [18]. All the mice were individually placed in a corner of an open field apparatus (L60 cm × W60 cm × H30 cm) just after the start of the dark cycle. The field was evenly illuminated at 20 lux. The open field was divided into a 30 cm ×30 cm central zone with a surrounding 15 cm-wide border zone. The mouse was allowed to explore freely in the open field for one hour, and the movement of the central point of its body was monitored with a CCD camera connected to a computer. The distance of ambulation and the percentage of time spent in the central zone were automatically calculated with Ethovision XT software (Brain Science Idea, Osaka, Japan). This test was repeated every 24 hours for three days.

To estimate the level of anxiety and spatial learning, an elevated plus maze test was employed [19], [20]. The maze consisted of two open arms (30 cm ×5 cm, no wall) and two closed arms (30 cm ×5 cm, surrounded by 15 cm high walls), which emerged from a central platform (5 cm ×5 cm) and was aligned perpendicularly. The apparatus was elevated 45 cm above the floor. All the mice were individually placed on the central area of the maze during the dark phase of the light/dark cycle, and allowed to move freely for 10 min. The movement of the mouse was monitored with a CCD camera connected to a computer. Time spent in the open arms, closed arms, and the central area were scored with Ethovision XT software.

The cliff-avoidance test and jumping events were evaluated as described previously [21], [22]. Briefly, tests were initiated by placing an animal onto a round platform (an inverted 20 cm high ×13.5 cm diameter glass cylinder). The time from an initial placement on the platform to falling down was recorded. If the animal remained to be on the platform after 7 min test, the jumping time was estimated as 7 min. Cumulative jumping events (%) were calculated according to the formula: (the number of falling animals/the number of total testing animal) ×100.

The forced swim test was carried out as described previously [23]. Briefly, mice were individually placed into a 20 cm high ×13.5 cm diameter glass cylinder filled to 12 cm of depth with water (23°C). Immobility floating time was measured for 3 min.

The prepulse inhibition (PPI) test was carried out as described previously [24], [25]. Each mouse was placed individually in a plexiglas tube mounted in a sound-attenuated chamber (Panlab, Barcelona, Spain). The startle responses of the mice were detected by a piezoelectric accelerometer. A computer was used to control the timing and presentation of acoustic stimuli and record the corresponding startle responses. The animals were placed into the chamber and allowed to habituate for 10 min under a presence of 65 dB white noise background. After habituation, the animals received 60 different trials (10 startle trials, 10 no-stimulus trials and 40 PPI trials) pseudo-randomly. The startle trial consisted of a single 120 dB white noise burst lasting 40 msec. The PPI trials consisted of a prepulse (20 msec burst of white noise with intensities of 69, 73, 77 or 81 dB), 100 msec later, followed by the startle stimulus (120 dB, 40 msec white noise). Each of prepulse trials was repeated 10 times. As to the no-stimulus trial, no stimulus was presented but the movement of the animal was scored. The result of the animal moving was measured during 100 msec after startle stimulus onset. PPI score for each prepulse level is expressed as % prepulse inhibition, calculated according to the formula: (1-(mean startle amplitude in prepulse trial/mean startle amplitude in startle trials)) ×100.

### Brain Histological Examination and Hematoxylin & Eosin (HE) Staining

Anesthetized five-week-old mice were perfused with PBS followed by 4% paraformaldehyde. The whole brain was removed from the skull and fixed for 16 h in 4% paraformaldehyde at 4°C and then washed in PBS, dehydrated, and embedded in paraffin for sectioning. Sections (10 μm thick) were stained with HE to examine the histological appearance of the brain under light microscopy [20]. To quantify the total cell number, hematoxylin positive cells were counted in 0.01-mm^2^ area of each brain section.

### Thionin Staining

For frozen sections, fixed brains were incubated in PBS containing 10% sucrose (pH 7.4). The buffer was changed three times over the next 24 hours. Then 8-μm sections of frozen brain were cut and mounted directly on APC-coated slides (Matsunami, Kishiwada, Japan). After drying, the brain sections were stained with 0.25% thionin solution (0.25% thionin, 0.55 μM lithium carbonate) [26].

### Immunohistochemical Staining

Paraffin-embedded sections were stained with antibodies against a neuronal marker NeuN (Chemicon, Temecula, CA, USA), astroglial marker glial fibrillary acidic protein (GFAP) (Cell Signaling Technology, Beverly, MD, USA), or neuroectodermal marker sex determining region Y-box 1 (Sox1) (Cell Signaling Technology) using the ABC kit according to the manufacturer's protocol (Vector Laboratories, Burlingame, CA, USA) [16]. NeuN- and GFAP-positive cells were counted in a 0.01-mm^2^ area of each brain section.

### Western Blotting and Densitometric Quantification

The brains of five-week-old mice were quickly removed, dissected and frozen in liquid nitrogen. Before the analyses, each whole brain was homogenized at 4°C in lysis buffer (0.1 M MES, pH 6.8, 0.5 mM MgSO_4_, 1 mM EGTA, 2 mM dithiothreitol, 0.75 M NaCl, 2 mM PMSF, 20 mM NaF, 0.5 mM sodium orthovanadate, 1 mM benzanidine, 25 mM β-glycerophosphate, 10 mM p-nitrophenylphosphate, 10 μg/ml aprotinin, 10 μg/ml leupeptin, and 1 μM okadaic acid). Sodium dodecyl sulfate-polyacrylamide gel electrophoresis (SDS-PAGE) and Western blot analysis were performed as described previously [16]. The anti-GFAP, anti-NeuN, anti-Sox1 and anti-β-actin antibodies were used in this study. The density of the each band was quantified using Image-J software, and normalized by β-actin.

### Statistical Analysis

Data are presented as mean ± S.D. Statistical analysis was performed using Graph Pad Prism 4.0 software (GraphPad, San Diego, CA, USA). Differences were determined by one-way analysis of variance (ANOVA) in the cliff-avoidance test and by the Log-rank test in the Kaplan-Meier survival curve. Means of variables in the other tests were compared by Student's *t* test.

## Results

### Generation and Confirmation of ZFP521 Mutant Mice

The mouse *ZFP521* gene was disrupted in ES cells using a targeting vector as shown in Fig. 1A. In this targeting vector, a 3.3-kb fragment containing most of exon 4 in *ZFP521* genomic DNA was replaced with neomycin resistance gene (neo). Simultaneously, an in-frame stop codon was newly inserted downstream of the remainder of exon 4. This deletion caused a loss of ZF2 to ZF30 motifs from ZFP521 protein, among which the ZF9 to ZF30 motifs are essential for ZFP521's neuralizing activity [10].

From the G418-resistant ES cell clones, correctly targeted clones were screened by PCR and confirmed by Southern blot analysis with the probe shown in Fig. 1A. Chimeric mice were generated by injection of targeted ES cells into blastocysts, and they were mated with C57BL/6J mice to obtain heterozygous mutant mice.

*ZFP521* heterozygous mutant mice (*ZFP521* ^+/Δ^) were successfully generated, and were phenotypically normal and fertile. *ZFP521* ^+/Δ^ mice were intercrossed, to generate homozygous mutant mice (*ZFP521* ^Δ/Δ^). Using Southern blot analysis (Fig. 1B) and DNA sequencing, we could confirm that exon 4 in the *ZFP521* gene was successfully disrupted in *ZFP521* ^Δ/Δ^ mice, and an in-frame stop codon was newly inserted downstream of the remainder of exon 4. Similarly, using Northern blot analysis, we were able to confirm that the expression of full-length ZFP521 mRNA was suppressed in the brain of *ZFP521* ^Δ/Δ^ mice (Fig. 1C).

### ZFP521^Δ/Δ^ Mice are Generated from Heterozygous Pairings at Expected Mendelian Ratios

*ZFP521* ^+/Δ^ mice were backcrossed onto C57BL/6J for more than 10 generations, and then intercrossed. From 15 *ZFP521* ^+/Δ^ breeding pairs, 142 pups were produced. They were genotyped immediately after birth, by PCR using genomic DNA extracted from the tail (Fig. 1D). Out of the pups examined, 36 were *ZFP521^+/+^*, 72 were *ZFP521* ^+/Δ^ and 34 were *ZFP521* ^Δ/Δ^, with a birth ratio consistent with Mendel's laws. Similarly, 71 were male and 71 were female, which was also consistent with the law (Table 1).

**Table 1:** Genotype analysis of 142 offspring derived from intercrossing of *ZFP521* ^+/Δ^ mice.

	+/+	+/Δ	Δ/Δ
Male	18	35	18
Female	18	37	16
Total	36	72	34

From 15 *ZFP521* ^+/Δ^ breeding pairs, 142 pups were produced. Note that the birth ratio of each genotype is consistent with Mendel's laws.

### Reduced Postnatal Growth of ZFP521^Δ/Δ^ Mice

At the time of birth, our *ZFP521* ^Δ/Δ^ pups had no obvious body defect, and were indistinguishable from *ZFP521^+/+^* and *ZFP521* ^+/Δ^ littermates. Their weight was not significantly different (*ZFP521^+/+^*: 2.10±0.09 g, *ZFP521* ^+/Δ^: 2.00±0.15 g, *ZFP521* ^Δ/Δ^: 1.88±0.06 g). However, *ZFP521* ^Δ/Δ^ mice showed significant weight reduction as they grew.

**Figure 2:**
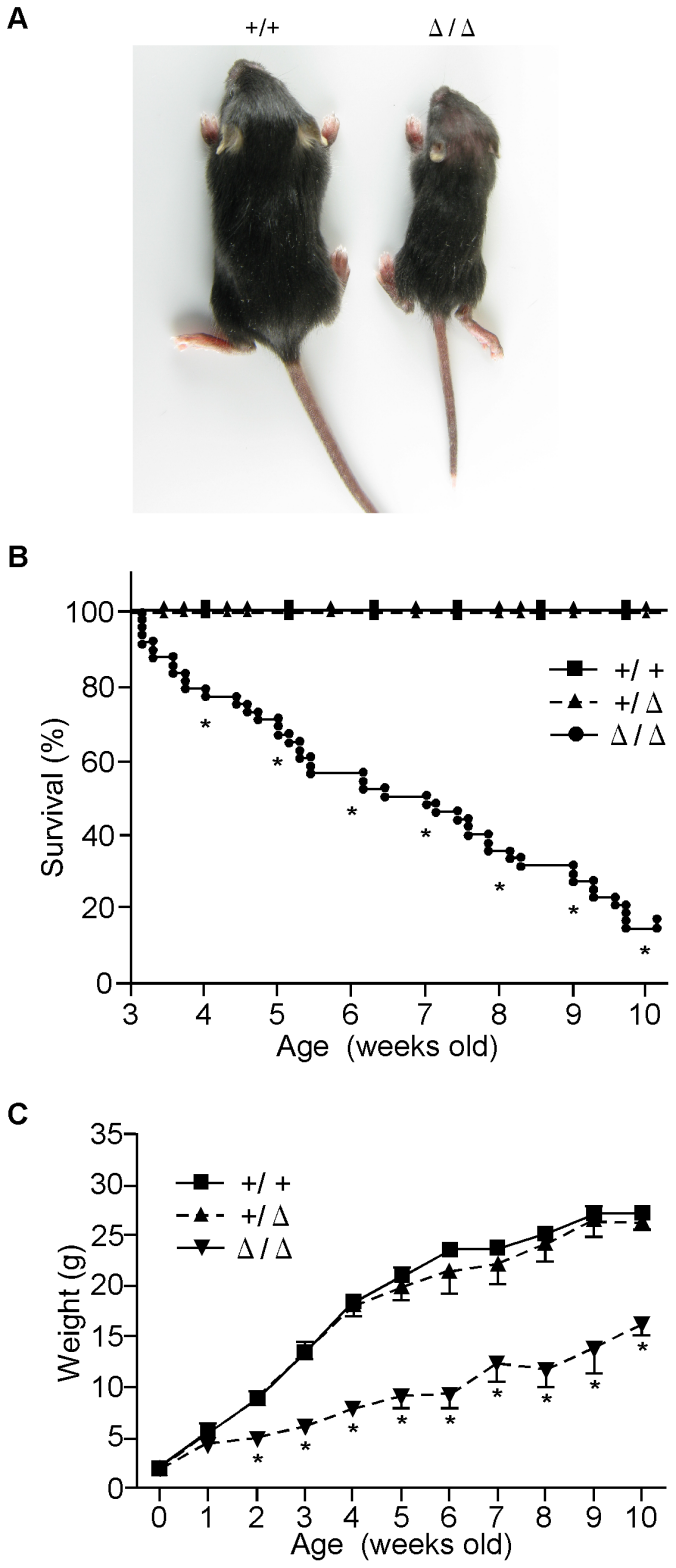
Growth and survival of mice. (A) Photograph of a two-week-old *ZFP521^Δ/Δ^* mouse and its *ZFP521^+/+^* littermate. (B) Growth curve of mice. Mice were housed singly, weighed weekly and. euthanized at a predetermined humane endpoint. ▪, *ZFP521^+/+^* mice; ▴, *ZFP521* ^+/Δ^ mice; ▾, *ZFP521* ^Δ/Δ^ mice. Each point with bar represents mean ± SD, n = 12. *, P<0.001, compared to *ZFP521^+/+^* mice. (C) Kaplan-Meier survival curves of mice. ▪, *ZFP521^+/+^* mice; ▴, *ZFP521^+/Δ^* mice; •, *ZFP521^Δ/Δ^* mice. *ZFP521^+/+^* mice, n = 114; *ZFP521^+/Δ^* mice, n = 181; *ZFP521^Δ/Δ^* mice, n = 48. *, P<0.001, in comparison with *ZFP521^+/+^* mice. Mice were monitored daily and euthanized at a predetermined humane endpoint.

As shown in Fig. 2A, the body size of two-week-old *ZFP521* ^Δ/Δ^ mice was obviously smaller than that of their *ZFP521* ^+/+^ littermates. The five-week-old *ZFP521* ^Δ/Δ^ mice weighed only about 50% as much as *ZFP521^+/+^* mice, and their weight was almost the same as that of 2-week-old *ZFP521^+/+^* mice (Fig. 2B). There was no change in this tendency of growth retardation after weaning. On the other hand, there was no significant difference in the weight from birth to 10 weeks of age between *ZFP521* ^+/+^ mice and *ZFP521* ^+/Δ^ mice. In addition to body weight, all organs of *ZFP521* ^Δ/Δ^ mice examined were significantly smaller and lighter than those of *ZFP521* ^+/+^ mice (Table 2). However, when examined in detail, it appeared that this weight difference varied according to organ type. For example, the thymus, spleen, adrenal gland and pituitary gland of *ZFP521^Δ/Δ^* mice were more than 40% lighter than those of the controls, while the heart and kidney were less than 30% lighter than those of the controls (Table 2). This suggested that ZFP521 might participate in the proliferation and differentiation of all cell types, although its degree of influence on development appeared to vary among organ types.

**Table 2:** Organ weights of 5-week-old *ZFP521* ^Δ/Δ^ mice.

	+/+		Δ/Δ	
	Weight (mg)	%	Weight (mg)	%
Body	19900±1270		11400±5700*	
Brain	356±83.5	2.76±0.166	237±55.3*	3.06±0.0751
Thymus	54.5±12.1	0.270±0.0666	26.0±7.98	0.230±0.0751
Lung	149±12.9	0.717±0.0669	96.6±6.33*	0.850±0.0200
Heart	123±7.60	0.590±0.0153	91.7±10.6	0.803±0.0484
Liver	1040±73.7	4.97±0.120	722±111	6.29±0.630
Kidney	161±9.35	0.770±0.0227	117±5.72*	1.03±0.0270
Spleen	63.0±7.44	0.303±0.0449	37.1±2.36*	0.330±0.0252
Adrenal gland	3.50±0.458	0.0317±0.0120	2.05±0.165*	0.0217±0.00167
Pituitary gland	2.13±0.0882	0.0107±0.000667	1.17±0.176*	0.0110±0.00100

Values are presented as mean ± S.D. n = 6.

*, p<0.05 compared with *ZFP521^+/+^* mice. Note that all organs of *ZFP521* ^Δ/Δ^ mice were significantly lighter than those of *ZFP521* ^+/+^ mice.

### Shortened Lifespan of ZFP521^Δ/Δ^ Mice

As *ZFP521* ^Δ/Δ^ mice showed an obviously shorter life-span than their wild-type littermates, we subjected them to Kaplan-Meier analysis (Fig. 2C). After we had determined the genotypes of the three-week-old mice, we followed the survivals of 48 *ZFP521* ^Δ/Δ^, 181 *ZFP521* ^+/Δ^ and 114 *ZFP521* ^+/+^ age-matched mice. The survival disadvantage of the *ZFP521* ^Δ/Δ^ mice was statistically significant from the first week, and this tendency for higher mortality did not change thereafter. Most of the *ZFP521* ^Δ/Δ^ mice died before 10 weeks after birth. The cause of death was unknown, and will require further investigation.

### Behavioral Analysis

*ZFP521* ^Δ/Δ^ mice showed abnormal behavior. They frequently stood on their hindlegs, and groomed themselves with their forelegs in the resting time. When a *ZFP521* ^Δ/Δ^ mouse was transferred to a cage where another mouse resided, it tended to spend a very long time following the resident mouse and constantly performed anogenital sniffing.

**Figure 3:**
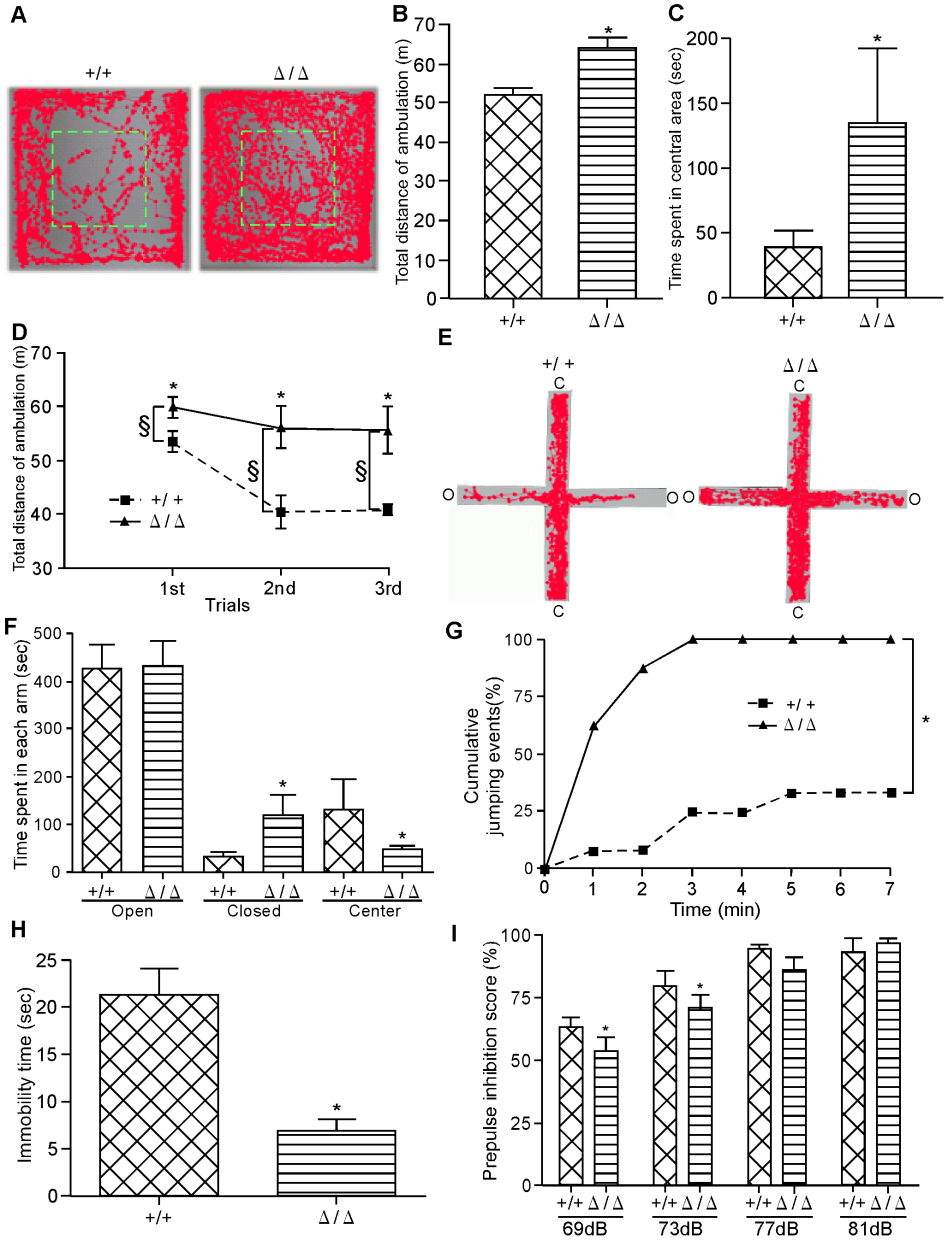
Behavioral analysis. Five-week-old *ZFP521* ^Δ/Δ^ mice and *ZFP521^+/+^* mice were used for all the analyses. (A–D) Open field test. (A) Representative locomotor tracks of *ZFP521* ^Δ/Δ^ mouse (right panel) and *ZFP521^+/+^* mouse (left panel) during 60 minutes. Green dashed box, central area of open field. (B) Total distance of ambulation of *ZFP521* ^Δ/Δ^ mice (right bar) and *ZFP521^+/+^* (left bar) mice during 60 min. Error bars indicate SD. n = 6. *, P<0.01, compared to *ZFP521^+/+^* mice. (C) Time spent in central area. *ZFP521* ^Δ/Δ^ mice spent more time in the central area of the open field than did *ZFP521^+/+^* mice during 60 minutes. Error bars indicate SD. n = 6. *, P<0.01, compared to *ZFP521^+/+^* mice. (D) Open field test was repeated three times every 24 hours. ▴, *ZFP521* ^Δ/Δ^; ▪, *ZFP521^+/+^*. Data represent mean ± SD. n = 6. *, P<0.01, compared to *ZFP521^+/+^* mice. Note that the total distance of ambulation in the second test was significantly shorter than that in the first test for *ZFP521* ^Δ/Δ^ mice. (E–F) Elevated plus maze test. (E) Representative locomotor tracks of *ZFP521^+/+^* mouse (left panel) and *ZFP521* ^Δ/Δ^ mouse (right panel) during 10 minutes. C, closed arm; O, open arm. (F) Duration time spent in closed arms, open arms, and central area. Error bars indicate SD. n = 6. *, P<0.05, compared to *ZFP521^+/+^* mice. Note that *ZFP521* ^Δ/Δ^ mice spent more time in the open arms than did *ZFP521^+/+^*, and less time in the central area. (G) Cliff-avoidance test. The cumulative frequency of jumping was determined. n = 5. *, P<0.01, compared to *ZFP521^+/+^* mice. Note that the cliff avoidance of *ZFP521* ^Δ/Δ^ mice was impaired. (H) Forced swim test. Immobility time was measured during the forced swim for 3 min. Data represent mean ± SD. n = 5 *, P<0.05, compared to *ZFP521^+/+^* mice. Note that the immobility time of *ZFP521* ^Δ/Δ^ mice was shorter than that of *ZFP521^+/+^* mice. (I) Prepulse inhibition test. The prepulse inhibition scores for each prepulse level were determined. Data represent mean ± SD. n = 5. *, P<0.05, compared to *ZFP521^+/+^* mice. Note that *ZFP521* ^Δ/Δ^ mice displayed significantly reduced inhibition at 69 dB and 73 dB prepulse, compared with *ZFP521^+/+^* mice.

Open field test was performed to examine locomotor activity and anxiety. The total distance of ambulation during an hour was significantly greater for *ZFP521* ^Δ/Δ^ mice than for control *ZFP521^+/+^* mice, suggesting that *ZFP521* ^Δ/Δ^ mice display a hyper-locomotive phenotype (Fig. 3A and 3B). *ZFP521* ^Δ/Δ^ mice spent significantly more time in the central zone than did control *ZFP521^+/+^* mice, suggesting that the anxiety level was lower in *ZFP521* ^Δ/Δ^ mice than in control (Fig. 3A and 3C). Other behavioral paradigms such as the elevated plus maze test confirmed a lowered anxiety level.

Interestingly, when this open field test was repeated three times every 24 hours, the total distance of ambulation in the second test was significantly shorter than that in the first test for *ZFP521^+/+^* mice, while it did not change significantly throughout the three tests for *ZFP521* ^Δ/Δ^ mice (Fig. 3D). This result suggests that learning and memory abilities might be impaired in *ZFP521* ^Δ/Δ^ mice.

In addition, elevated plus maze test was performed to examine the anxiety level (Fig. 3E and 3F). *ZFP521* ^Δ/Δ^ mice spent a significantly longer time in the open arms and a shorter time in the central area than did *ZFP521* ^+/+^ mice. There was no difference in the time spent in the closed arms between *ZFP521* ^Δ/Δ^ mice and *ZFP521* ^+/+^ mice. These results suggest that the *ZFP521* ^Δ/Δ^ mice had a lower anxiety level than *ZFP521* ^+/+^ mice.

The cliff-avoidance test was used to assess maladaptive impulsive rodent behavior (Fig. 3G). *ZFP521* ^+/+^ mice tended to explore the edge of the platform and then remained on the platform until the end of the test. By contrast, *ZFP521* ^Δ/Δ^ mice demonstrated marked hyper-locomotion throughout the test session and peering down behavior. In contrast to more than 60% of the *ZFP521* ^+/+^ mice that remained on the platform after 7 min, all of the *ZFP521* ^Δ/Δ^ mice were peering down within 3 min.

Forced swimming was used to test for depressive-like behavior (Fig 3H). It has been reported that the immobility time of some model mice with depressive disorder is significantly prolonged in comparison with wild-type mice [27]. Conversely, the immobility time of our *ZFP521* ^Δ/Δ^ mice was significantly shorter than that of *ZFP521^+/+^* mice.

The prepulse inhibition test of the startle response is an index of the inhibitory function of a weak sensory stimulus (prepulse), and is one of the major tests for schizophrenic behavior. In this test, the *ZFP521* ^Δ/Δ^ mice displayed prepulse inhibition scores at 69 dB and 73 dB that were significantly lower than those of *ZFP521^+/+^* mice (Fig. 3I).

### Histopathological Analysis of Brain

**Figure 4:**
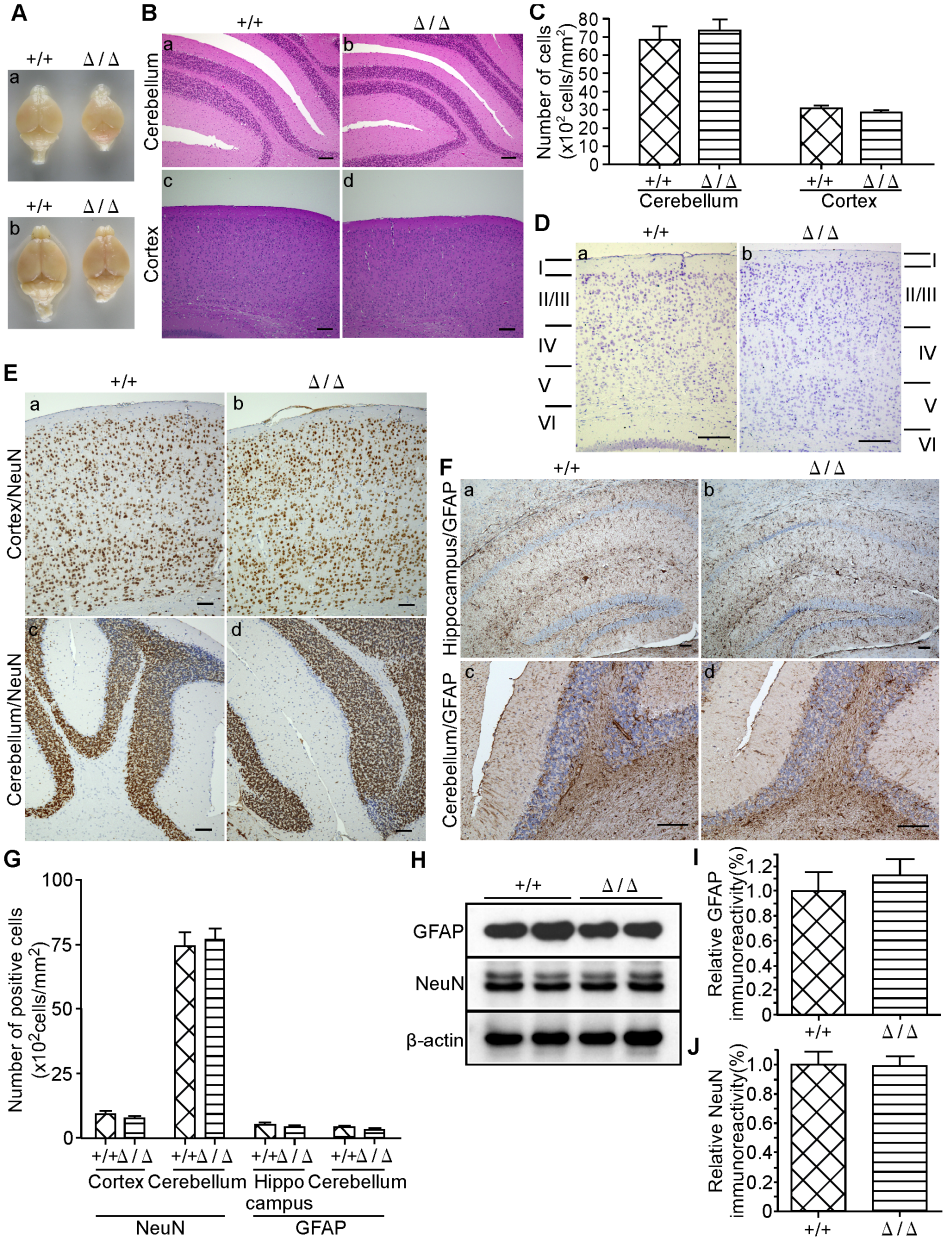
Histopathological analysis of the brain. (A) Photographs of the whole brains of two-week-old (a) and twenty-week-old (b) mice. The brain of *ZFP521* ^Δ/Δ^ mouse (right brain) was smaller than that of *ZFP521^+/+^* littermate (left brain). (B) HE-stained sections of cerebellum and cerebral cortex of five-week-old mice. Bars  = 100 μm. a and b, cerebellum; c and d, cerebral cortex; a and c, *ZFP521^+/+^* mice, b and d, *ZFP521* ^Δ/Δ^ mice. Note that the cerebellum and cerebral cortex of *ZFP521* ^Δ/Δ^ mice appeared histologically normal. (C) Quantification of the number of cells in cerebellum and cerebral cortex of five-week-old mice. We counted hematoxylin positive cells, and statistically analyzed. Data represent mean ± SD. n = 5. Note that there was no significant difference in the number of cells between *ZFP521^+/+^* mice and *ZFP521* ^Δ/Δ^ mice. (D) Cortical cell layers in five-week-old mice. Sagittal sections of cerebral cortex from *ZFP521^+/+^* mouse (a) and *ZFP521* ^Δ/Δ^ mouse (b) were stained with thionin. Bars  = 100 μm. Note that the formation of six neural cell layers in the cerebral cortex of *ZFP521* ^Δ/Δ^ mice appeared normal. (E) Immunohistochemical detection of neurons in five-week-old mice. Cerebral cortex (a and b) and cerebellum (c and d) of *ZFP521^+/+^* mouse (a and c) and *ZFP521* ^Δ/Δ^ mouse (b and d) were immunohistochemically stained with anti-NeuN antibody. Bars  = 100 μm. Note that no significant abnormality, including the number, size and positioning of differentiated neurons, was found. (F) Immunohistochemical detection of astroglial cells in five-week-old mice. Hippocampus (a and b) and cerebellum (c and d) of *ZFP521^+/+^* mouse (a and c) and *ZFP521* ^Δ/Δ^ mouse (b and d) were immunohistochemically stained with anti-GFAP antibody. Bars  = 100 μm. Note that no abnormality of astroglial cells was found. (G) Quantification of the number of anti-NeuN and anti-GFAP positive cells in cerebellum, dentate gyrus and cerebral cortex of five-week-old mice. Data represent mean ± SD. n = 6. Note that there was no significant difference in the number of cells between *ZFP521^+/+^* mice and *ZFP521* ^Δ/Δ^ mice. (H) Immunoblot detection. Immunoblots of lysates from the cerebellum of five-week-old *ZFP521^+/+^* and *ZFP521* ^Δ/Δ^ mice were probed with anti-GFAP (upper panel) and anti-NeuN antibody (middle panel). Anti-β-actin antibody (bottom panel) was used as control for protein loading. The experiment was repeated at least three times, and all results were similar. A representative result is shown. (I) Densitometric quantification of GFAP protein in the cerebellum of five-week-old mice. The density of the GFAP band shown above was quantified using Image-J software, and normalized by β-actin. The vertical axis indicates the quantified density of GFAP protein relative to that in *ZFP521^+/+^* mice. Error bar indicates SD. n = 6. Note that there was no significant difference between *ZFP521^+/+^* mice and *ZFP521* ^Δ/Δ^ mice. (J) Densitometric quantification of NeuN protein in the cerebellum of five-week-old mice. The density of the NeuN band shown above was quantified using Image-J software, and normalized by β-actin. Error bar indicates SD. n = 6. Note that there was no significant difference between *ZFP521^+/+^* mice and *ZFP521* ^Δ/Δ^ mice.

The whole brains of two-week-old (a) and twenty-week-old (b) *ZFP521* ^Δ/Δ^ mice were smaller than those of *ZFP521^+/+^* mice (Fig. 4A). The shapes of the cerebellum and cerebral cortex of *ZFP521* ^Δ/Δ^ mice were morphologically normal, and HE-stained sections of both brain regions also showed no abnormality (Fig. 4B). The numbers of hematoxylin-positive cells in the granular layer of the cerebellum and layer II of the cerebral cortex were counted (Fig. 4C). There was no significant difference in cell density between *ZFP521^+/+^* and *ZFP521* ^Δ/Δ^ mice, in both parts of the brain. In addition to HE-staining, we also employed thionin-staining to clarify the neuronal cell layer, in order to confirm that the six neural cell layers in the cerebral cortex of *ZFP521* ^Δ/Δ^ mice had formed normally (Fig. 4D).

Next, we immunohistochemically stained the cerebral cortex and cerebellum of *ZFP521* ^Δ/Δ^ mice with an antibody against NeuN, which is a specific marker of differentiated neurons [28], [29], but no significant abnormality in the number, size or positioning of differentiated neurons was found (Fig. 4E and 4G). Nor could we find any abnormality of astroglial cells in the hippocampus (Fig. 4F, a and b) and cerebellum (Figure 4F, c and d) of *ZFP521* ^Δ/Δ^ mice, using an antibody against GFAP, which is a specific marker of astrocytes [30], [31]. In addition to immunohistochemical studies, immunoblot analyses with anti-GFAP and anti-NeuN antibodies were performed to quantify the levels of proteins in the cerebellum of five-week-old mice (Fig. 4H–J). This revealed no significant differences in the expression levels of both GFAP and NeuN between *ZFP521^+/+^* and *ZFP521* ^Δ/Δ^ mice.

**Figure 5:**
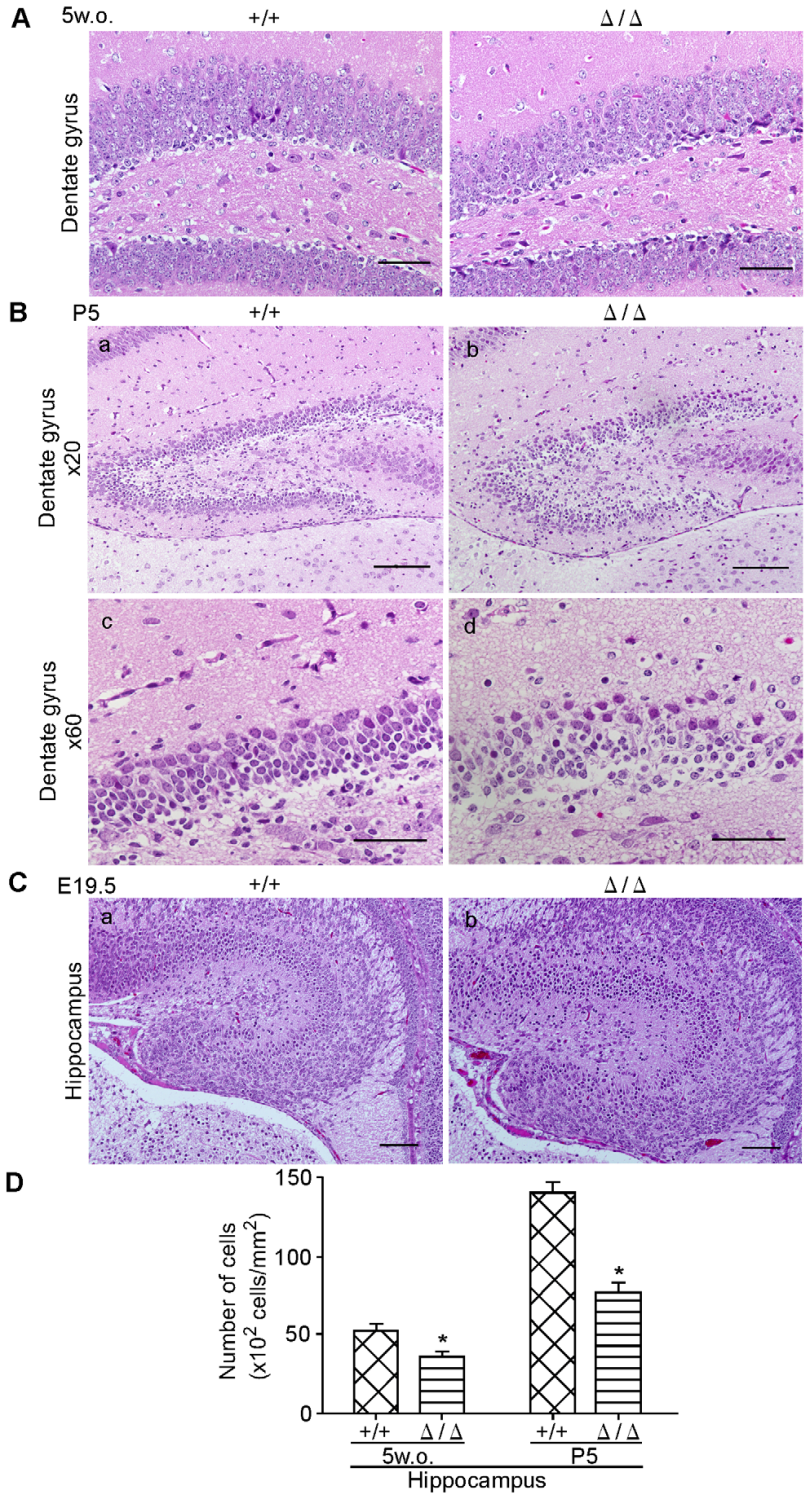
HE-staining of dentate gyrus at each developmental stage. (A) HE-staining of dentate gyrus in five-week-old mice. Bars  = 50 μm. a, *ZFP521^+/+^* mouse; b, *ZFP521* ^Δ/Δ^ mouse. Note that the density of neuronal cells was obviously lower and the neuronal cell layer was more obscure in *ZFP521* ^Δ/Δ^ mouse. (B) HE-staining of dentate gyrus in five-day-old mice. Bars  = 100 μm (a and b) and 50 μm (c and d). a and c, *ZFP521^+/+^* mouse; b and d, *ZFP521* ^Δ/Δ^ mouse. Note that the number of neuronal cells appear smaller in five-day-old *ZFP521* ^Δ/Δ^ mouse than in *ZFP521^+/+^* mouse. (C) HE-staining of dentate gyrus and hippocampus in E19.5 embryos. Bars  = 100 μm. a, *ZFP521^+/+^* embryo; b, *ZFP521* ^Δ/Δ^ embryo. Note that the dentate gyrus and hippocampus of the *ZFP521* ^Δ/Δ^ embryo were poorly organized, and the number of cells in the neuronal cell layer was smaller in the *ZFP521* ^Δ/Δ^ embryo than in the *ZFP521^+/+^* embryo. (D) Quantification of the number of cells in dentate gyrus. Hematoxylin positive cells were counted. Error bar indicates SD. n = 6. *, P<0.05, compared to *ZFP521^+/+^* mice. Note that the number of cells in dentate gyrus of both five-week-old (5w.o) and five-day-old (P5) mice were significantly decreased compared with *ZFP521^+/+^* littermates. Error bar indicates SD. n = 6.

The dentate gyrus in the hippocampus of five-week-old *ZFP521* ^Δ/Δ^ mice was also morphologically normal, but granular neurons were clearly reduced in number and the border of the granular cell layer was indistinct compared with that in control *ZFP521^+/+^* mice (Fig. 5A and 5D). In the dentate gyrus of five-day-old *ZFP521* ^Δ/Δ^ mice, the number of granular neurons was smaller than that in *ZFP521^+/+^* mice (Fig. 5B and 5D). We further examined the brain of E19.5 mice (Fig. 5C). At this stage, the hippocampus of the *ZFP521* ^Δ/Δ^ brain was poorly organized, whereas it was well organized in *ZFP521^+/+^* mice. These results suggest that formation of the neuronal cell layers in the hippocampus and dentate gyrus of E19.5 *ZFP521* ^Δ/Δ^ embryos was affected, and that the neural cells may still have been in the process of migrating to the appropriate region.

Taken together, the results of histopathological analysis suggest that ZFP521 is concerned with formation of the neuronal cell layers of the dentate gyrus in the hippocampus.

### Detection of Undifferentiated Neural Precursor Cells in ZFP521^Δ/Δ^ Brain

ZFP521 is reported to be essential for driving the intrinsic neural differentiation of mouse ES cells *in vitro* [10]. If so, the behavioral abnormalities of *ZFP521* ^Δ/Δ^ mice might be caused not only by the morphological abnormality of the dentate gyrus and hippocampus, but also by suppression of transition of ES cells to neuronal cells.

**Figure 6:**
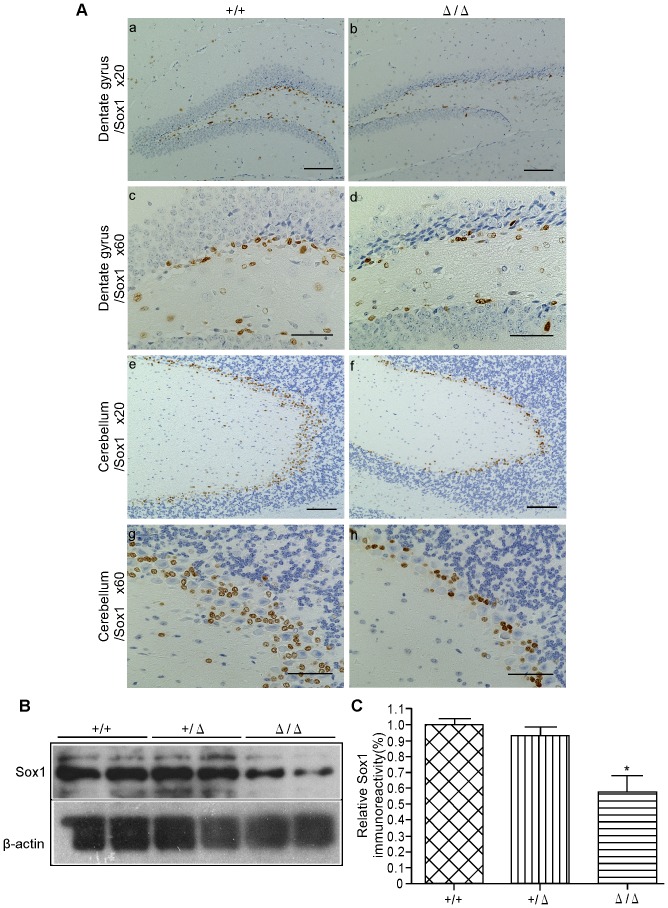
Detection of neuroectodermal and neural stem cells. (A) Immunohistochemical detection of neuroectodermal and neural stem cells. Sections of the dentate gyrus (a–d) and cerebellum (e–h) from five-week-old *ZFP521^+/+^* mouse (a, c, e, g) and *ZFP521* ^Δ/Δ^ mouse (b, d, f, h) were stained with anti-Sox1 antibody. Note that the number of Sox1-positive cells in dentate gyrus and cerebellum of *ZFP521* ^Δ/Δ^ mice appears smaller than that in *ZFP521^+/+^* mice. Bars  = 100 μm (a, b, e, f) and 50 μm (c, d, g, h). (B) Immunoblot detection. Immunoblots of lysates from the cerebellum of five-week-old *ZFP521^+/+^*, *ZFP521* ^+/Δ^, and *ZFP521* ^Δ/Δ^ mice were probed with anti-Sox1 antibody (upper panel). Anti-β-actin antibody (bottom panel) was used as control for protein loading. The experiment was performed at least three times, and all results were similar. A representative result is shown. Note that Sox1 expression was decreased in the cerebellum of *ZFP521* ^Δ/Δ^ mice. (C) Densitometric quantification of Sox1 protein in the cerebellum of five-week-old mice. The density of the Sox1 band shown above was quantified using Image-J software, and normalized by β-actin. The vertical axis indicates the quantified density of Sox1 protein relative to that in *ZFP521^+/+^* mice. Error bar indicates SD. n = 6. *, P<0.001, compared to *ZFP521^+/+^* mice. Note that Sox1 expression was significantly decreased in the cerebellum of *ZFP521* ^Δ/Δ^ mice compared with *ZFP521^+/+^* mice.

We examined the presence of neural progenitor cells in the dentate gyrus and cerebellum of five-week-old *ZFP521* ^Δ/Δ^ mice, using a Sox1-specific antibody. This revealed fewer Sox1-positive cells in the dentate gyrus and cerebellum of *ZFP521* ^Δ/Δ^ mice than those in *ZFP521^+/+^* mice (Fig. 6A). Similarly, immunoblotting with the antibody demonstrated that Sox1 expression was significantly decreased in the cerebellum of five-week-old *ZFP521* ^Δ/Δ^ mice (Fig. 6B and 6C). These results suggest that *ZFP521* is involved in the differentiation of ES cells to neural progenitor cells, also *in vivo*.

## Discussion

We generated mice lacking exon 4 of the *ZFP521* gene, and having an in-frame stop codon inserted downstream of the remainder of exon 4. As a result, ZF2-30 were deleted from ZFP521 protein in *ZFP521* ^Δ/Δ^ mice.

**Figure 7:**
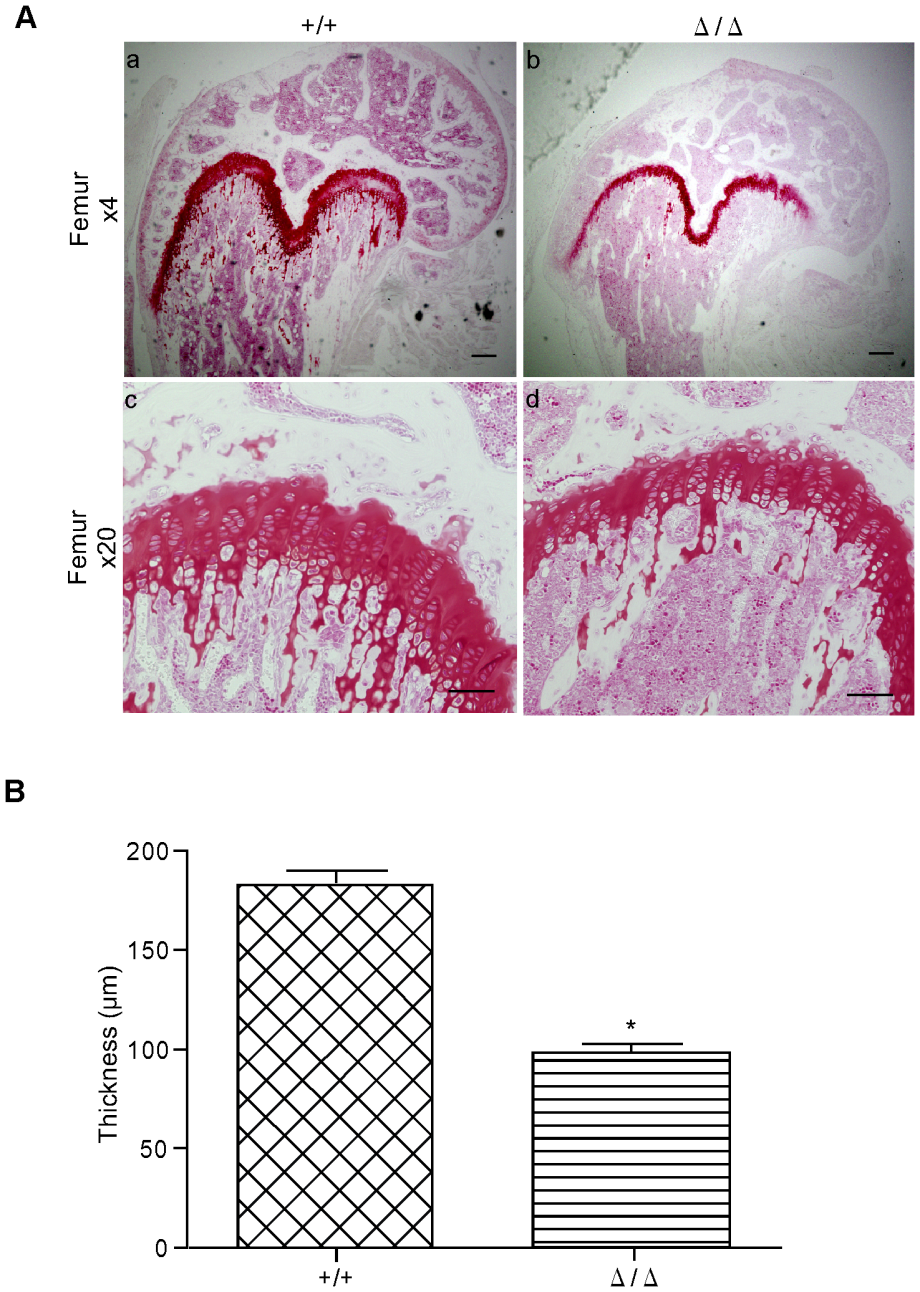
Histological analysis of the femoral growth plate. (A) Femoral growth plate from five-week-old mice, stained with safranin O solution. The growth plate layer is strongly stained. Bars  = 100 μm (a and b) and 50 μm (c and d). a and c, *ZFP521^+/+^* mouse; b and d, *ZFP521* ^Δ/Δ^ mouse. Note that the growth plate of *ZFP521* ^Δ/Δ^ femur appears thinner than that of *ZFP521* ^+/+^. (B) Histomorphometric analysis of thickness of femoral growth plate. Error bars indicate SD. n = 22. *, P<0.001. Note that the thickness of the growth plate of *ZFP521* ^Δ/Δ^ femur is decreased by 54%, compared with that of *ZFP521* ^+/+^ femur.

Seriwatanachai, *et al.* originally generated chondrocyte-specific *ZFP521*-deficient mice, and using them, they reported that ZFP521 is required downstream of PTHR1 signaling to act on chondrocyte proliferation and differentiation [7], [32], [33]. In our *ZFP521* ^Δ/Δ^ mice, the thickness of the femoral growth plate was decreased by 54%, compared with that in *ZFP521* ^+/+^ (Fig. 7). Our result is in agreement with that of Seriwatanachai, *et al.* and confirms that our mutant mice were successfully generated.

Kamiya, *et al.* reported that ZFP521 is essential and sufficient for driving the intrinsic neural differentiation of mouse ES cells. Using cultured ES cells and shRNA-mediated gene knockdown techniques, they clearly demonstrated that *ZFP521*-depleted ES cells do not undergo neural conversion but tend to halt at the epiblast stage *in vitro*. They also demonstrated that deletion of ZF9-30 from ZFP521 protein resulted in loss of its neuralizing activity [10]. Based on these findings, we expected that *ZFP521* ^Δ/Δ^ mice would be embryonically lethal. However, contrary to our expectation, the mice were viable, and their birth ratio was consistent with Mendel's laws. This might have been due to the difference in the experimental system employed; Kamiya, *et al.* performed their experiments *in vitro*, whereas ours were done *in vivo*. The existence and induction of a compensatory gene for *ZFP521 in vivo* is suspected. On the other hand, our results are partly consistent with those of Kamiya, *et al.* in that the number of Sox1-positive neural progenitor cells was lower in the brain of our *ZFP521* ^Δ/Δ^ mice.

Kang, *et al.* have also generated and analyzed *ZFP521*-deficient mice, and reported recently that Zfp521 acts as a key regulator of adipose commitment and differentiation *in vitro* and *in vivo* [6]. As their homozygous *ZFP521*-deficient mice died shortly after birth, they used E18.5 embryos for their analysis. On the other hand, our *ZFP521* ^Δ/Δ^ mice lived longer, but most of them died before 10 weeks after birth. It seems unlikely that an eating disorder would have been the cause, because the tendency for growth retardation remained constant during and after weaning. The cause of death in our *ZFP521^Δ/Δ^* mice remains unknown, and is a matter for further investigation.

The results of the open field test suggested that *ZFP521* ^Δ/Δ^ mice have a hyper-locomotive phenotype (Fig. 3A and 3B). Hyper-locomotion is a classical feature of rodent models of schizophrenia and corresponds to the psychomotor agitation evident in schizophrenic patients [34], [35]. The results of the open field test (Fig. 3C), elevated plus maze test (Fig. 3E and 3F), cliff-avoidance test (Fig. 3G) and forced swim test (Fig. 3H) suggested that *ZFP521* ^Δ/Δ^ mice have a significantly lower anxiety level and a higher impulsivity level. The prepulse inhibition of startle response is one of the major tests for schizophrenic behavior. Deficits in prepulse inhibition are observed in patients suffering from schizophrenia, and other psychiatric disorders [36], [37] and can be induced in animals by administration of various schizophrenomimetics [38], [39], [40]. Our *ZFP521* ^Δ/Δ^ mice displayed significantly reduced inhibition at 69 dB and 73 dB prepulse (Fig. 3I). These results strongly suggest that *ZFP521* ^Δ/Δ^ mice have schizophrenia-relevant symptoms.

The hippocampus is likely to be an impacted region in schizophrenic patients [41], [42]. In patients with schizophrenia, as well as model mice, histopathological alteration in the dentate gyrus of the hippocampus has been reported. There, neural stem cell proliferation and adult hippocampal neurogenesis in the dentate gyrus were decreased [43], [44]. The results of histopathological studies of our *ZFP521* mutant mice were consistent with these reports. In our *ZFP521* ^Δ/Δ^ mice, Sox1-positive neural stem cells in the dentate gyrus were significantly reduced in number (Fig. 6A). Furthermore, in the dentate gyrus of five-day-old and five-week-old *ZFP521* ^Δ/Δ^ mice, the border of the granular cell layer was indistinct and granular neurons were clearly reduced in number compared with those in control *ZFP521^+/+^* mice (Fig. 5A, 5B and 5D). At the E19.5 stage, the hippocampus of *ZFP521* ^Δ/Δ^ embryos was poorly organized, whereas it was well organized in *ZFP521^+/+^* embryos (Fig. 5C). Therefore, the abnormal behavior of *ZFP521* ^Δ/Δ^ mice may be due to such histopathological abnormalities in the dentate gyrus.

Many neurons of the adult brain are generated prenatally, but in the hippocampus, cerebellum, and olfactory bulb, neurons are also formed in postnatal life. This occurs through the proliferation and differentiation of adult neural stem cells, which exist at two locations under normal conditions: the subventricular zone (SVZ) of the lateral ventricles and the subgranular zone (SGZ) of the dentate gyrus in the hippocampus. Newborn neurons in the SGZ migrate a short distance into the granular cell layer of the dentate gyrus, and integrate into the existing neural circuitry [45]. Lack of ZFP521 does not prevent, but may attenuate, the transition of epiblast stem cells into Sox1-positive neural stem cells, or otherwise suppress the proliferation of slow-dividing stem cells within the SGZ, which results in a reduced number of granular neurons and an indistinct border of the granular cell layer of the dentate gyrus.

Mice lacking exons 2 and 3 of the disrupted-in-schizophrenia (DISC1) gene displayed schizophrenia-relevant behaviors, similarly to our *ZFP521* mutant mice [46]. DISC1 is a promising candidate gene for susceptibility to schizophrenia, and is involved in newborn neuron migration in the SGZ [47], [48]. Although the molecular mechanism underlying the pathophysiological function of DISC1 still remains elusive, many proteins were found to be involved in the pathway [49], [50]. ZFP521 may also be involved in the pathway.

Taken together, these findings indicate that ZFP521 directly or indirectly affects formation of the neuronal cell layers of the dentate gyrus in the hippocampus, and as a result, *ZFP521* ^Δ/Δ^ mice display schizophrenia-relevant symptoms. *ZFP521* ^Δ/Δ^ mice may be a useful animal model for schizophrenia research.
